# Recent Advances in Low-Carbon Membrane Materials: A Review of Material Development and Application Research

**DOI:** 10.3390/membranes16040120

**Published:** 2026-03-30

**Authors:** Meixuan Xin, Huamei He, Feifei Wei, Xia Zheng, Yuan Xiang

**Affiliations:** 1 College of Materials and Chemistry & Chemical Engineering, Chengdu University of Technology, Chengdu 610059, China; 2024050625@stu.cdut.edu.cn (H.H.); 2024050637@stu.cdut.edu.cn (F.W.); zhengxia@stu.cdut.edu.cn (X.Z.); 2 College of Physics, Chengdu University of Technology, Chengdu 610059, China

**Keywords:** low-carbon membrane materials, coupled membrane separation process, separation mechanisms, application progress

## Abstract

Traditional membrane separation materials suffer from drawbacks such as a high carbon footprint, significant energy consumption, membrane fouling, and the potential for secondary pollution. Under the dual drivers of carbon neutrality and carbon peak strategies, as well as the deepening of environmental governance, low-carbon membrane separation materials have emerged as a pivotal direction for the green transformation of membrane technology, leveraging their core advantages of green raw materials, low-energy preparation, and high application adaptability. This green transition is primarily achieved through the development of green raw materials and preparation processes, the enhancement of separation efficiency, and a reduction in operational energy consumption. Consequently, this review systematically summarizes the low-carbon design principles, key performance metrics, separation mechanisms, catalytic coupling technologies, and the recent application progress of several mainstream types of low-carbon membrane materials. It further identifies current bottlenecks in the research of low-carbon membrane materials such as performance trade-offs, challenges in scalable fabrication, and long-term operational instability. Finally, the review proposes future research directions aimed at developing novel membrane materials that integrate low-carbon attributes, excellent separation performance, and multifunctionality.

## 1. Introduction

Under the global context of advancing sustainable development and green industrial transformation, separation processes, as one of the four core processes in the chemical industry, are inevitably undergoing a low-carbon transition [1,2,3]. Membrane separation technology has been widely applied in wastewater treatment, gas separation, and resource extraction owing to its high efficiency, operational simplicity, and energy-saving characteristics [4]. At present, membrane separation technology still excessively relies on fossil energy and involves production processes with high energy consumption [5]. In addition, the inherent permeability and selectivity trade-offs and membrane fouling significantly constrain separation efficiency. This requires either higher operating pressure or more complex multistage treatment processes to alleviate the problem, which in turn increases the carbon footprint and environmental burden [6]. Therefore, the development of novel low-carbon membrane materials that combine high permeability, precise selectivity, and long-term stability is critical to advancing the green transition [7].

To simultaneously enhance separation efficiency and low-carbon attributes, the following strategies have been primarily adopted: (1) reducing carbon footprints at the source through the utilization of low-carbon materials, green solvents, and low-energy fabrication processes; (2) decreasing energy consumption via low-pressure and low-temperature operational modes; (3) endowing membrane materials with excellent antifouling properties and superior stability, thereby reducing the additional energy consumption caused by frequent chemical cleaning and short service life; and (4) integrating unit operations through process coupling to improve overall treatment efficiency [8,9,10,11]. For instance, Iulianelli et al. [12] fabricated dense polylactic acid (PLA) membranes from low-carbon raw materials such as corn starch and sugarcane using an evaporation-induced low-energy technique. This approach not only substantially reduced carbon emissions but also overcame the intrinsic performance limitations of conventional bio-based polymers, thereby broadening the operational window and representing a significant step toward sustainable biopolymer membranes. Similarly, Zhang et al. [13] developed an anion exchange membrane–membrane bio-membrane reactor (AEM-MBfR) system by coupling AEM with a bioreactor. Through electrostatic interaction mechanisms and enzymatic catalysis, efficient nitrate removal was achieved, while catalytic and denitrifying microbial communities were integrated within the membrane matrix to enhance stability and selectivity. Despite these advances, the unified realization of high efficiency, low energy consumption, large-scale production, and sustainability remains challenging in practical applications [14]. Consequently, the development of low-carbon membrane materials is essential for meeting the low-carbon demands of industrial wastewater treatment, gas separation, and resource extraction [15].

**Figure 1:**
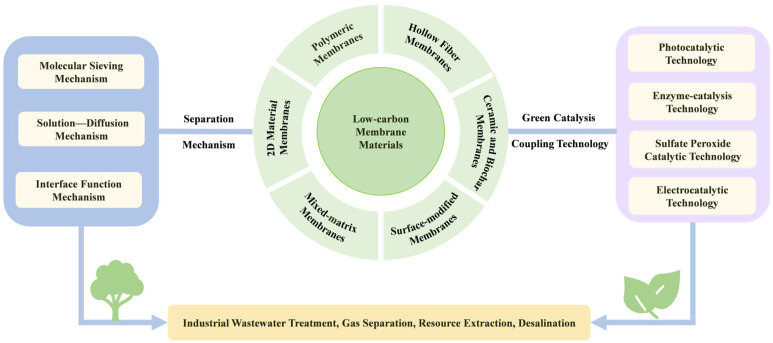
Schematic illustration of the overall framework and conceptual roadmap.

Therefore, this review centers on low-carbon membrane materials and systematically summarizes the low-carbon design strategies, green fabrication routes, and performance characteristics of polymer membranes, two-dimensional material membranes, mixed-matrix membranes, ceramic membranes, and biochar membranes. This review also explains the synergistic effects achieved by integrating these materials with catalytic technologies. It analyzes the core separation mechanisms of low-carbon membranes and explores their applications in key areas such as wastewater treatment, gas separation, and resource recovery. Current technical challenges are discussed, and an outlook on future development trends is provided. This aims to serve as a reference for promoting the comprehensive green transformation and sustainable development of membrane separation technology. The overall framework of this review is illustrated in Figure 1.

## 2. Categories of Low-Carbon Membrane Separation Materials

Low-carbon membrane materials can be categorized into polymeric membranes, two-dimensional (2D) material membranes, mixed-matrix membranes, surface-modified membranes, ceramic membranes, and biochar membranes based on their composition and structural characteristics. Each category employs distinctive low-carbon designs and fabrication strategies, along with unique physicochemical properties which can reduce carbon emissions while maintaining separation performance [16]. The core advantages and major limitations of each type of low-carbon membrane material are summarized in Table 1.

### 2.1. Low-Carbon Polymeric Membranes

Polymeric membranes remain the most widely utilized materials in separation technologies owing to their cost-effectiveness, ease of processing, and scalability [44]. One of the most effective strategies for realizing the green transition of membrane materials is the utilization of renewable biopolymers or recycled waste materials as alternatives to traditional petroleum-based polymers [45,46]. Among these, many biopolymers inherently contain reactive functional groups, including ester groups in polyesters and hydroxyl (-OH) or amino (-NH_2_) groups in polysaccharides, which facilitate further chemical modification and structural regulation [47,48]. For instance, Khamwichit et al. [49] prepared cellulose acetate membranes derived from coconut biomass via microbial cultivation and applied them to CO_2_/CH_4_ separation. A selectivity of 35.52 was achieved at 0.28 MPa, demonstrating that the biomass-derived polymers could provide competitive separation performance under moderate conditions.

**Figure 2:**
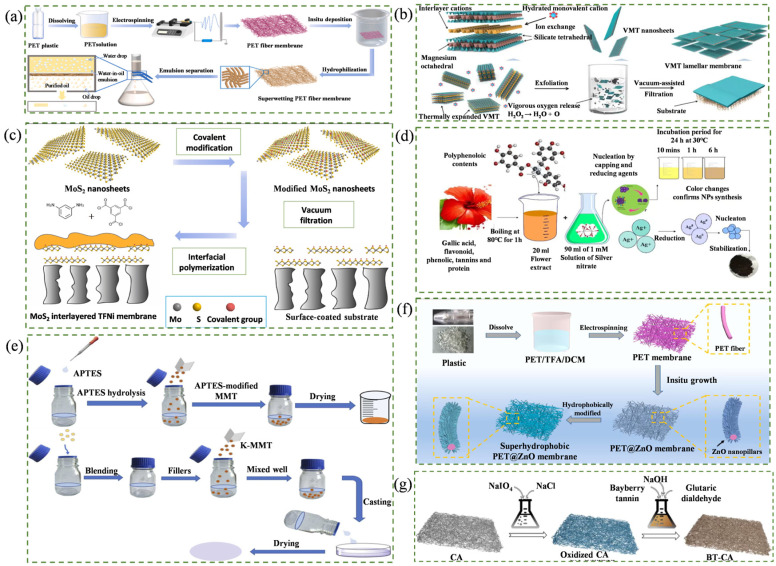
Schematic fabrication process of low-carbon membrane separation materials. (**a**) Fabrication of PET fiber membrane [51]; (**b**) fabrication of 2D VMT membrane [59]; (**c**) MoS_2_ interlayered TFNi membrane [60]; (**d**) fabrication of PES/AgNPs MMM [61]; (**e**) fabrication of Pebax/K-MMT MMM [62]; (**f**) fabrication of PET@ZnO membrane [63]; (**g**) fabrication of BT-CA membrane [64].

Waste-derived membranes represent another important pathway for carbon reduction [18]. Discarded plastics such as polyethylene terephthalate (PET); polyethylene (PE); polyvinyl chloride (PVC); and polystyrene (PS), and spent membrane materials (e.g., polyamide (PA); polysulfone (PSF); polyethersulfone (PES); and polyvinylidene fluoride (PVDF)) can be converted into new separation membranes through physical reprocessing or chemical regeneration [50]. Although waste-derived membranes often exhibit satisfactory stability, secondary processing may compromise their mechanical strength. Chen et al. [51] proposed a “waste control by waste” strategy by extracting PET from plastic bottles and fabricating fibrous membranes via electrospinning. The resulting membranes exhibited a dense yet interconnected structure suitable for oil–water separation. Under gravity-driven conditions alone, a separation efficiency of 99% was achieved, effectively mitigating plastic-related environmental pollution. The fabrication process is illustrated in Figure 2a.

In addition, green membrane fabrication processes can be beneficial for the low-carbon strategy. The non-solvent-induced phase separation (NIPS) uses water instead of solvents for evaporation, and it is currently the most widely used membrane fabrication process [52]. Among them, the solubility and performance of the polymer in the solvent are the key factors affecting the membrane separation performance [53]. Common polar organic solvents (such as N,N-dimethylformamide (DMF), N,N-dimethylacetamide (DMAc), etc.) are generally toxic and volatile, causing serious environmental impacts [54]. Green solvents include biogenic or low-toxicity solvents (such as ethyl lactate and γ-valyl alcohol ketone), which can significantly reduce the carbon footprint and environmental pollution during the preparation process [55,56,57]. However, the solubility and performance of the polymer in the solvent are relatively low, which may have adverse effects on the structure and separation performance of the membrane material. Therefore, the development of green solvents and the optimization of process conditions are the current key research directions. Xie et al. [58] used 5-(dimethylamino)-2-methyl-5-oxovaleric acid methyl ester as a green solvent and employed the NIPS method to prepare PVC membranes. These membranes had a uniform pore structure, and the pure water permeation flux exceeded 5000 L·m^−2^·h^−1^·bar^−1^. Moreover, they exhibited excellent selectivity, with a retention rate of 98% for sodium alginate. This achievement represents a dual breakthrough in the application of green solvents and membrane performance.

### 2.2. Low-Carbon Two-Dimensional Material Membranes

Two-dimensional membranes, (including graphene oxide (GO), MXene, two-dimensional metal–organic frameworks (MOFs), and g-C_3_N_4_ have attracted considerable attention due to their ultrathin nanoporous structures, high specific surface areas, and tunable interlayer channels [21,65,66,67]. From a low-carbon perspective, their development emphasizes the use of naturally abundant layered minerals, biomass-derived carbon materials, and environmentally benign preparation routes [68,69]. Green solvents such as water, bioethanol, low-toxicity ionic liquids, and deep eutectic solvents, combined with low-energy techniques, including ultrasonic exfoliation, ball milling, and electrochemical intercalation, can contribute to reducing fabrication-related carbon emissions [70,71]. Tian et al. [59] assembled a layered vermiculite (VMT) membrane derived from natural silicate minerals. The membrane exhibited excellent stability in aqueous, organic, and extreme pH environments, enabling efficient dye removal while mitigating swelling issues commonly encountered in 2D membranes. The preparation route is shown in Figure 2b.

However, during the preparation process of 2D membranes, surface defects are prone to occur, and when operating under extreme high-pressure conditions, the upper separation layer may undergo phenomena such as separation, compression, and expansion, which is not conducive to the separation efficiency and service life of the membranes [22,72]. Recently, studies have shown that by using a covalent crosslinking method, covalent chemical bonds can be formed between 2D materials, thereby enhancing the stability of the membranes [73,74]. Wang et al. [60] prepared carboxylated (MoS_2_-COOH) and amidated (MoS_2_-CONH_2_) molybdenum disulfide nanosheets through covalent modification, and constructed TFNi-MoS_2_-COOH and TFNi-MoS_2_-CONH_2_ film nanocomposite membranes. The results showed that the covalently modified 2D membranes had a stable separation structure, with the retention rates of ethyl viologen reaching 90.0% (MoS_2_-COOH) and 87.1% (MoS_2_-CONH_2_) and maintained stable separation performance during the 72 h test. The preparation route is shown in Figure 2c.

In addition, the biomimetic mineralization interlayer strategy can also enhance the stability of the membrane. Inspired by sandwich-like interlayer structures, an inorganic mineral interlayer grown in situ with an ordered structure can form high activity between the separation layer and the supporting layer. This organic–inorganic hybrid structure not only enables precise control of the pore structure but also enhances the compressive strength [75,76]. Li et al. [77] used the waste cocoon shell as the substrate, with PVA/CS as the upper-layer structure, and grew a mineral composite layer composed of hydroxyapatite (HAP) and titanium dioxide (TiO_2_) in situ between the two layers. They successfully prepared the HAP/TiO_2_@PVA/CS composite membrane; this membrane had excellent hydrophilicity and mechanical strength, while in the oil–water separation application, its retention rate was as high as 98.98%. Moreover, the TiO_2_ in the interlayer had photocatalytic properties. The HAP/TiO_2_@PVA/CS membrane had a photocatalytic degradation rate of 92.83% for methyl blue, demonstrating excellent antibacterial performance, achieving a synergistic enhancement of the integration of membrane structure and function.

The introduction of bio-based nanoparticles rich in functional groups (e.g., -OH, -COOH), such as cellulose, tannic acid, and amino acid derivatives, into 2D nanomaterials has proven effective [24,78]. These nanoparticles can not only form an antifouling hydrophilic layer on the surface but also act as supporting components intercalated between the layers, thereby enabling precise regulation of the interlayer spacing and enhancing mechanical properties [23]. Arshad et al. [79] successfully fabricated functional MoS_2_ nanomaterials by employing non-toxic L-cysteine as a sulfur source during the synthesis of MoS_2_ nanosheets, followed by hydrophilic modification with L-DOPA and hydrophilic 2D ions. The resulting materials were subsequently immobilized onto the surface of nanofiltration membranes. This hydrophilic modification of MoS_2_ effectively enhanced the membrane permeability and antifouling performance, offering a promising strategy for achieving multifunctionality in 2D material membranes.

Furthermore, compared with traditional membrane fabrication methods, such as vacuum assisted filtration and layer-by-layer assembly, interfacial polymerization (IP) can produce 2D material membranes with more stable structures [80,81]. Through interface regulation at the molecular level, the functional groups on the surface of 2D materials (such as -O, -OH, -F) can be used to control the material transport within the membrane [82,83]. In thin film nanocomposite (TFN) membranes, 2D materials participate in forming the polyamide layer, creating channels with lower water transmission resistance. While in TFNi membranes, 2D materials act as the intermediate layer, which can regulate monomer diffusion and result in thinner and defect-free polyamide separation layers [84]. This molecular scale design not only enhances the structural stability of the membrane but also enables the membrane to simultaneously have high throughput and high selectivity [11]. Huang et al. [85] introduced positively charged amino-terminated MXene as an intermediate layer during the interface polymerization process, and precisely controlled the diffusion behavior of the amine monomer (piperazine). They successfully prepared ultrathin polyamide composite membranes (TFN AM). The results showed that the thickness of these membranes were six times thinner compared to the original polyamide membrane. Moreover, with the introduction of MXene, the surface of the membrane gained a higher positive charge density, enhancing the Donnan repulsion effect and resulting in a selectivity of 17.5 for Li^+^/Mg^2+^ in the TFN AM membrane. And its performance remained stable even after continuous operation for 100 h, providing a simple and effective strategy for efficient extraction of lithium resources from salt lake brines.

### 2.3. Low-Carbon Mixed-Matrix Membranes

Mixed-matrix membranes (MMMs) are membrane materials fabricated by integrating two or more materials within a matrix [86]. Low-carbon MMMs are typically constructed by incorporating green or multifunctional fillers into a low-carbon polymer matrix [87]. Their low-carbon design strategy primarily relies on the selection of environmentally friendly substrates and fillers, as well as green preparation methods [88]. By uniformly dispersing porous nanofillers (e.g., MOFs, carbon nanomaterials, and zeolites) into renewable or recyclable polymer matrices (e.g., cellulose, chitosan, and waste plastics), and by adopting fabrication procedures that minimize solvent consumption and energy input, the carbon footprint associated with membrane production can be substantially reduced [28,89,90]. Bashir et al. [61] synthesized AgNPs fillers using rose extract and incorporated them into PES to fabricate PES/AgNP MMMs. The AgNPs regulated polymer-chain packing and nanoscale architecture, thereby endowing the membranes with strong desalination performance, dye removal capability, and antifouling resistance. Notably, complete rejection (100%) was achieved for both methylene blue and methyl orange. The fabrication process is illustrated in Figure 2d.

Interfacial compatibility and filler functionality remain decisive factors governing MMM performance [27]. Jiang et al. [62] improved the compatibility between inorganic montmorillonite (MMT) and the Pebax matrix through silane functionalization, and subsequently prepared Pebax/K-MMT MMMs via in situ curing. This integrated design, combining filler modification with interfacial optimization, yielded an outstanding CO_2_/N_2_ selectivity of 162.4. The fabrication process is illustrated in Figure 2e. In addition, the employment of low-toxicity, biodegradable solvents provides an effective route to reduce carbon emissions during membrane fabrication. Chai et al. [91] prepared GO/CA membranes via NIPS using ethyl lactate as a green solvent. Owing to the favorable affinity between the ethyl lactate ester groups and CA, GO was uniformly dispersed within the CA matrix, leading to increased hydrophilicity (by 14.51%) and porosity (by 47.4%). Consequently, the pure water flux and COD rejection improved by factors of 2.56 and 3.46, respectively.

### 2.4. Low-Carbon Surface-Modified Membranes

Surface-modified membranes are obtained through low-energy and low-pollution physical or chemical treatments that tailor membrane surfaces to enhance separation performance, stability, and antifouling resistance to align with green and low-carbon goals [92]. Based on the modification pathway, they are generally classified as surface coating and surface grafting [32,93]. Within low-carbon design frameworks, surface coating typically replaces petroleum-derived modifiers with green coating materials compatible with low-carbon membrane matrices, such as chitosan, lignin nanoparticles, and tannic acid [29]. These coatings are deposited by dip-coating, spraying, or vapor deposition to form ultrathin, strongly adherent functional layers [31,94]. In contrast, surface grafting generates reactive sites on base membranes via low-energy initiation methods (including plasma treatment, chemical grafting, and enzyme-catalyzed grafting) and subsequently polymerizes functional monomers containing target groups (e.g., -NH_2_, -COOH, -SO_3_H) to form grafted chains on the surface [30,95].

Hybrid modification strategies combining crosslinking/curing with in situ growth have become increasingly prominent [96,97]. By employing crosslinkers to reinforce reactions between the functional materials (or grafted monomers) and the membrane surface, such approaches retain mild processing conditions, low energy demands, and a high stability, all while simultaneously delivering improved separation performance, cycling durability, and antifouling resistance [33,98]. Therefore, they represent an important pathway toward the low-carbon upgrading of membrane separation technologies [99,100]. Xiong et al. [63] utilized waste plastic bottles as a feedstock and grew hydrophilic ZnO nanoparticles in situ on the membrane surface, producing a PET@ZnO membrane with a superhydrophilic surface and underwater superoleophobicity. In oil–water separation, the membrane achieved 99.6% efficiency under gravity-driven conditions and exhibited no discernible performance decay over ten consecutive cycles. This durability was attributed to the strong interactions between the ZnO nanostructure and the PET substrate, which enhanced mechanical and chemical stability. The fabrication process is illustrated in Figure 2f. In a related study, Luo et al. [64] used plant-derived tannin as a crosslinker to fabricate cellulose–tannin (BT-CA) membranes for uranium extraction from seawater. Coordination of the interactions between ester groups and uranyl ions conferred high adsorption stability, providing insights into low-carbon interfacial engineering (Figure 2g).

### 2.5. Low-Carbon Ceramic and Biochar Membranes

**Figure 3:**
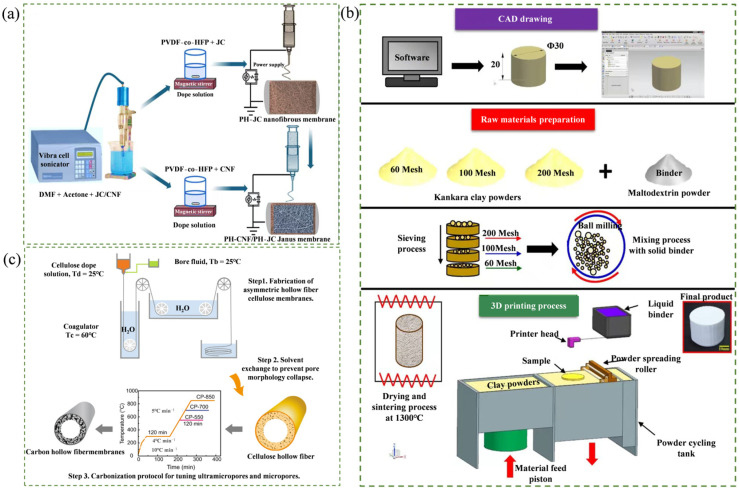
Schematic fabrication process of low-carbon membrane separation materials. (**a**) Fabrication of PH-CNF/PH-JC Janus membranes [106]; (**b**) fabrication of clay-based ceramic membranes (via 3D printing technology) [108]; (**c**) fabrication of CP-850 HFMs [41].

Ceramic membranes, as representative inorganic membrane materials, exhibit excellent thermal and chemical stability, enabling long-term operation under harsh environments such as high temperatures and strongly acidic or alkaline conditions [36,101]. In low-carbon fabrication, ceramic membranes can be produced using industrial waste residues containing Al_2_O_3_, ZrO_2_, or SiC (e.g., fly ash and coal gangue) or natural minerals (e.g., kaolin and clay) when combined with low-temperature or microwave-assisted green sintering processes [34,102,103]. These methods can produce membranes that possess high permeability, long service lives, and good recyclability [37,104]. Sandhya et al. [105] successfully prepared ceramic membranes using an inexpensive clay, and these ceramic membranes achieved a 70.4% COD removal rate in the treatment of wastewater from natural rubber, demonstrating the potential of low-carbon ceramic membranes in practical applications. Hwa et al. [106] used natural clay powder as a raw material and utilized 3D printing technology to successfully prepare porous ceramic membranes with good mechanical properties. In the COD removal test, the removal rate of this membrane reached 97.78%, indicating the feasibility of preparing ceramic membranes using 3D printing technology in practical applications. The fabrication process is illustrated in Figure 3a.

Biochar membranes are porous carbon-based separation membranes derived from renewable biomass feedstocks (such as straw, sawdust, nutshells, algae, and biopolymers) via pyrolysis or activation [38,39,40]. These membranes offer advantages including low costs and renewable precursors [107]. Hossain et al. [107] developed a PH-CNF/PH-JC dual-layer membrane using jute carbon (JC) and carbon nanofibers (CNFs). This bilayer design synergistically combined the high surface area and electrical conductivity of JC with the hydrophilicity of CNFs, resulting in excellent long-term stability in seawater. The membrane delivered a high flux of 78.42 kg·m^−2^·h^−2^ and maintained a NaCl rejection rate of 99.98% after 50 h of operation. The fabrication process is illustrated in Figure 3b.

### 2.6. Low-Carbon Hollow Fiber Membranes

The hollow fiber membranes (HFMs) are constructed by assembling a large number of hollow tubular fibers and feature excellent packing density [109,110]. This tubular self-supporting structure can effectively reduce the equipment’s floor space and material consumption, while also mitigating foulant deposition [111]. HFMs exhibit excellent low-carbon characteristics, antifouling capabilities, and backwashing performance, effectively alleviating the environmental impact and operating costs [42]. Moreover, compared with the traditional spiral winding process, dry–wet spinning combined with the interface polymerization process can significantly reduce the mass transfer resistance of HFMs [112]. Lei et al. [41] used microcrystalline cellulose and ionic liquids as raw materials, and, through the method of dry–wet spinning combined with high-temperature carbonization, prepared a CHFM-850 membrane. This membrane had an excellent and stable pore structure, and exhibited a high hydrogen permeability of 148.2 GPU. It also showed high selectivity for H_2_/CO_2_ and H_2_/N_2_, with values of 83.9 and 800, respectively. Furthermore, this device could operate stably for over 120 h under extreme conditions of 10 Pa pressure, 90 °C temperature, and 100% relative humidity, providing a sustainable high-performance solution for hydrogen purification based on renewable raw materials. The preparation process is shown in Figure 3c.

However, the low selectivity on the surface of HFMs has always been a major challenge [43]. Studies have shown that using coating and interface processes could effectively alleviate this issue [113]. Teber et al. [114] used a low-toxicity solvent, dimethyl sulfoxide (DMSO), and a combined dry–wet spinning and polydimethylsiloxane (PDMS) coating method to prepare a PSF hollow fiber membrane substrate, which was then applied to air separation. The results showed that after three coating cycles adding 3% PDMS, this membrane achieved an O_2_/N_2_ selectivity of 4.70 and an O_2_ permeability of 8.49 GPU.

## 3. Catalytic–Membrane Coupling for Low-Carbon Separation-Degradation Integration

Under the targets of “carbon peak and carbon neutrality,” standalone membrane separation processes often face persistent challenges, including high transmembrane pressure requirements, membrane fouling, notable carbon footprints, and limited capability for treating concentrated or refractory pollutants [115]. These issues constrain their ability to meet the demands of green development. To address such bottlenecks, separation catalysis-integrated designs have been increasingly adopted as a core strategy [116]. Building on green preparation concepts from MMMs and surface-modified membranes, catalytic technologies, including photocatalysis, enzymatic catalysis, persulfate activation, and electrocatalysis, are coupled with membrane separation to achieve the synergistic effect of “1 + 1 > 2” [117,118]. Such integration provides a green pathway to reduce energy consumption, mitigate fouling, extend membrane lifetime, and enhance advanced purification performance [119].

### 3.1. Photocatalysis–Membrane Separation Coupling

**Figure 4:**
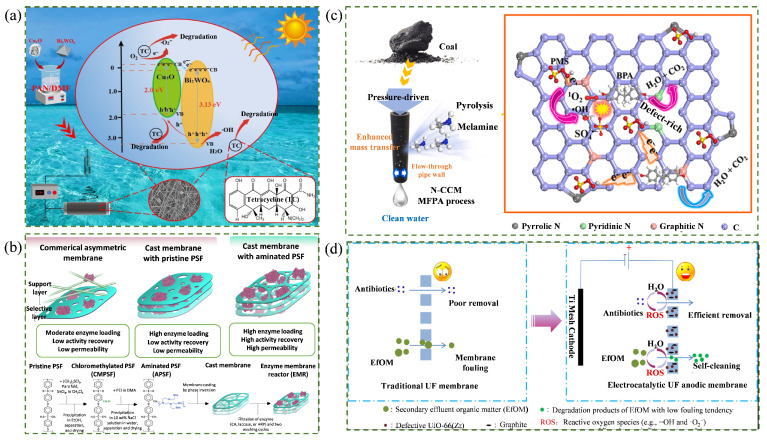
Coupling mechanisms of low-carbon membrane materials with catalytic technologies. (**a**) Photocatalysis [129]; (**b**) enzymatic catalysis [130]; (**c**) PMS catalysis [131]; (**d**) electrocatalysis [132].

Photocatalysis is widely regarded as a sustainable and efficient catalytic route and has been extensively integrated with membrane separation [120,121]. Under light irradiation and appropriate pH conditions, semiconductors such as TiO_2_, ZnO, and g-C_3_N_4_ can be directionally incorporated into membrane matrices [122,123,124]. Photogenerated charge carriers subsequently react with dissolved oxygen and water to generate reactive oxygen species (ROS), including hydroxyl radicals (·OH) and superoxide anion radical (·O_2_^−^), enabling in situ degradation of pollutants enriched near the membrane interface [125,126]. This coupling not only improves the removal efficiency of pollutants but also alleviates fouling and short service lives, thereby reducing overall energy demand and carbon footprints [127,128]. Guo et al. [129] fabricated Bi_2_WO_6_/Cu_2_O@PAN nanofiber membranes by co-electrospinning Bi_2_WO_6_/Cu_2_O photocatalysts with PAN. This configuration combined catalytic activity with the high specific surface area and facile recyclability of nanofiber membranes. Under visible light irradiation, degradation efficiencies of 90.87% and 85.12% were achieved for chlortetracycline and tetracycline, respectively, mainly attributed to ·O_2_^−^ generated by the photocatalyst. Notably, the activity remained stable after six cycles, supporting its practical applicability. The catalytic mechanism is illustrated in Figure 4a.

### 3.2. Enzyme Catalysis–Membrane Separation Coupling

Enzyme catalysis is considered a green and efficient approach due to mild reaction conditions and high selectivity [133]. Enzymes such as laccase, tyrosinase, and peroxidase exhibit strong compatibility with bio-based membrane materials [134]. By employing immobilization strategies (such as adsorption, encapsulation, crosslinking, and covalent grafting) enzymes can be integrated into membranes while reducing the reliance on organic solvents [135,136,137,138]. Moreover, the substrate specificity of enzymes enables targeted degradation or transformation of specific pollutants during membrane operation [139,140]. Popkov et al. [130] immobilized carbonic anhydrase (CA) and horseradish peroxidase (HRP) onto aminated PSF membranes. While maintaining membrane permeability, catalytic activity increased by 6 and 36 times for CA and HRP, respectively. HRP retained 97.5% of its initial activity after five consecutive cycles. This work not only demonstrated efficient immobilization but also addressed key issues of enzyme-catalytic membranes, including enzyme deactivation and mass transfer limitations. The fabrication process and advantages are illustrated in Figure 4b.

### 3.3. Persulfate Activation–Membrane Separation Coupling

Persulfate activation refers to the activation of peroxymonosulfate (PMS) or peroxydisulfate (PDS) to generate strong oxidizing species (including sulfate radical (SO_4_^·−^), ·OH, ·O_2_^−^, and singlet oxygen (^1^O_2_)), thereby mineralizing stable and refractory organic pollutants into CO_2_ and H_2_O [141,142]. Chemical activation is most commonly used, with bases, transition metals, and carbon-based materials serving as typical activators [143]. This route offers broad pH adaptability, high oxidation capacity, and strong degradation efficiency [144]. Coupling persulfate activation with membrane separation can reduce the energy demand of standalone membrane processes, enhance deep purification capability, and mitigate the impact of concentrated pollutants on both membrane materials and the environment [145,146]. Ma et al. [131] fabricated a nitrogen-doped catalytic ceramic membrane (N-CCM) by impregnating coal-based carbon with melamine as a nitrogen source, followed by high-temperature pyrolysis. Nitrogen doping introduced defect structures that served as active sites and tuned the electron distribution of the carbon matrix. Through synergistic adsorption by the N-CCM and ROS generation from PMS activation, a bisphenol A (BPA) degradation efficiency of 98.3% was achieved. The proposed mechanism is illustrated in Figure 4c.

### 3.4. Electrocatalysis–Membrane Separation Coupling

Electrocatalytic membrane systems employ conductive membranes (e.g., carbon-based, porous titanium-based, Magnéli-phase, and ceramic membranes) as electrodes, enabling synergistic pollutant removal through electrostatic adsorption, electrochemical oxidation and reduction, and physical retention under an applied electric field [147,148,149]. When membranes serve as anodes, oxidative capacity is inversely related to the oxygen evolution reaction (OER) overpotential. Direct electro-oxidation proceeds via electron transfer, whereas indirect oxidation produces ROS through electrolyte-mediated reactions, enabling pollutant degradation [150,151,152]. Zhou et al. [132] utilized a defective UiO-66 (Zr) anode membrane characterized by low OER overpotential. The ROS generated during electro-oxidation removed over 96.6% of antibiotics and achieved 100% bacterial inactivation in municipal wastewater. Moreover, under a low current density, fouling was completely eliminated. This work established an electrocatalytic membrane with integrated “offensive and defensive” functionality, overcoming the dual limitations of conventional membranes fouling susceptibility and catalytic electrode deactivation. The mechanism is illustrated in Figure 4d.

## 4. Separation Mechanisms of Low-Carbon Membrane Materials

The separation mechanisms of traditional membrane materials mainly include molecular sieving, solution diffusion, and interfacial interaction [153]. However, due to the integration of green design in low-carbon membranes, their separation methods have become more diverse and complex [154]. To ensure the separation performance of low-carbon membranes, their separation mechanisms are usually achieved through the collaborative action of multiple separation mechanisms [155].

### 4.1. Molecular Sieving Mechanism

**Figure 5:**
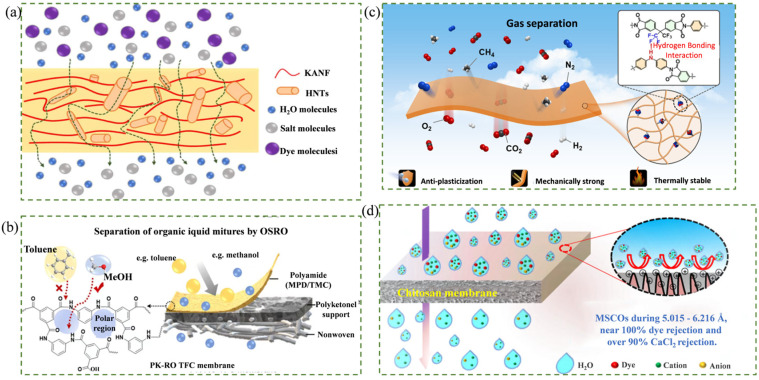
Separation mechanisms of low-carbon membrane materials. (**a**) Molecular sieving [162]; (**b**) solution–diffusion [163]; (**c**,**d**) interfacial interactions [164,165].

For porous membranes, molecular sieving represents a fundamental separation principle [156,157]. This mechanism relies on the differences in the kinetic diameters of components, achieved by precisely regulating the pore size and architecture of the membrane to dimensions intermediate between the target molecules and impurities, thereby enabling effective separation [158,159]. In low-carbon membrane systems, 2D material membranes, biochar membranes, and MMMs based on nanofillers and bio-based polymers primarily operate via this mechanism. Channel regulation in such membranes is commonly achieved through surface functionalization of fillers or interlayer pillaring strategies [160,161]. Xu et al. [162] employed halloysite nanotubes (HNTs) and nanofibers (KANF) with distinct morphologies to modulate pore size and geometry. During separation of an EBT/NaCl mixture, the membrane achieved an EBT rejection of 99% while maintaining a NaCl rejection of only 10% relative to the pristine KANF membrane, demonstrating enhanced sieving precision and alleviating limitations of single-filler systems. The mechanism is illustrated in Figure 5a.

### 4.2. Solution–Diffusion Mechanism

Solution–diffusion is the dominant mechanism for dense polymeric membranes. The process involves two consecutive, essentially irreversible steps: dissolution (adsorption at the upstream surface) and diffusion (mass transfer through the membrane) [166,167,168]. Separation performance is governed by the differences in solubility and diffusivity of components within the membrane, which are determined by the material’s intrinsic properties [169]. However, many bio-based polymers exhibit characteristics such as high solubility but low diffusivity, which can lead to limited membrane flux and a tendency to swell in water [170,171]. These limitations can be mitigated through asymmetric rigid monomer incorporation, crosslinking, or grafting strategies [172,173]. Liu et al. [163] fabricated a solvent-resistant polyketone-reverse osmosis (PK-RO) composite membrane using aliphatic polyketone (PK) as the support and a highly crosslinked polyamide (MPD/TMC) selective layer formed via interfacial polymerization. When applied to organic solvent reverse osmosis, the membrane exhibited high flux and excellent selectivity for mixtures such as methanol/toluene—notably, a NaCl rejection up to 97% was achieved. The mechanism is illustrated in Figure 5b.

### 4.3. Interfacial Interaction Mechanism

Interface mechanisms refer to the processes by which active sites on the surface or within the pores of membrane materials differentially interact with feed components through physical (e.g., hydrogen bonding, electrostatic interactions, π−π stacking, van der Waals forces) or chemical interactions (e.g., coordination, covalent bonding) [174,175,176]. These interactions enable the preferential adsorption and enrichment of target molecules, followed by their efficient separation via diffusion and desorption [177,178,179]. Adsorption is the core step, and selectivity can be enhanced by introducing groups with strong affinity toward target species (such as -NH_2_, -OH, -F, -COOH, metal coordination sites, and charged moieties) into the membrane structure [180,181,182,183,184]. Xiao et al. [164] synthesized a diamine acid (HCBDA) containing -NH- groups based on carbazole and polymerized it with polyimide to prepare HCB-PI permeation membranes. The introduced -NH- groups enhanced the hydrogen-bond interactions and adsorption of gaseous pollutants, achieving of selectivity of 101 and 32.3 with H_2_/CH_4_ and CO_2_/CH_4_, respectively. The mechanism is illustrated in Figure 5c. In another study, Long et al. [165] exploited electrostatic interactions by fabricating renewable chitosan (CS) membranes via solution coating. Due to abundant positive charges within and on the surface, the effective rejection of anions (e.g., SO_4_^2−^, Cl^−^) and dyes was achieved (CaCl_2_ rejection: 94.8%; dye rejection: >99%). Hydrophilic -OH groups also maintained stable water flux above 5 LMH. The mechanism is shown in Figure 5d.

## 5. Applications of Low-Carbon Membrane Materials

With increasingly strict requirements for environmental protections, low-carbon membrane materials exhibit good environmental compatibility, stable operation performance, and efficient selective separation ability [185]. These materials have shown broad application prospects in industrial wastewater treatment, gas separation, resource recovery, and other related fields [186,187].

### 5.1. Industrial Wastewater Treatment

With ongoing advances in textile printing and dyeing, electroplating, and coal chemical industries, industrial wastewaters containing heavy metals, refractory organics, and high salinity often exhibit complex composition, poor biodegradability, and toxicity [188,189,190]. Low-carbon membrane materials, supported by green design and catalytic coupling, have emerged as attractive alternatives to conventional membranes [191,192].

For dye removal, Foroutan et al. [193] constructed a microalgae-based composite membrane by coating microalgal extracts onto membrane surfaces, achieving methylene blue rejection above 90% with strong antifouling performance. For heavy metal removal, Karim et al. [194] introduced cellulose nanocrystals onto cellulose membranes to fabricate fully bio-based membranes with high flux, achieving removal efficiencies of 86% (Cu^2+^), 74% (Fe^2+^/Fe^3+^), and 100% (Ag^+^) via adsorption by the functional cellulose layer. Catalytic coupling further can enable the efficient removal of emerging contaminants such as tetracycline (TC). Yu et al. [195] reported a flexible, porous, mechanically robust Fenton-like fiber membrane (FeOOH-CFM), achieving 99% TC degradation through ROS generated by PMS activation, providing a closed-loop solution for refractory organics. Low-carbon membranes are also effective for oil–water separation. Hu et al. [196] fabricated microfiltration membranes from natural cellulose in aqueous solvent systems, featuring uniform submicron pores. Without complex chemical modification, these membranes achieved >99.9% rejection and high-flux separation for oil-in-water nanoemulsions with droplet sizes below 200 nm. Collectively, these cases demonstrate the strong potential of low-carbon membranes for scalable industrial wastewater treatment.

### 5.2. Gas Separation

As a core operation in energy, environmental, and chemical engineering sectors, gas separation urgently requires low-energy and high-efficiency upgrading under carbon neutrality objectives [197,198]. Low-carbon membranes, benefiting from low-pressure operation, phase-change-free processing, and scalability, show promise in CO_2_ capture, H_2_ purification, biogas upgrading, and air separation [199,200].

Tseng et al. [201] fabricated high-density gas separation membranes from waste tire rubber via hot pressing. The membranes exhibited CO_2_/N_2_ selectivity above 12 and showed negligible selectivity variation after 60 days, indicating strong environmental durability and offering a route toward low-cost carbon-capture membranes. Kukobar et al. [202] designed a MXene-wrapped apatite (G-HAP) membrane, achieving high H_2_/CH_4_ selectivity and permeability at a low pressure by utilizing interfacial voids between MXene and HAP. Prasad et al. [203] incorporated graphene nanoparticles into chitosan/silk fibroin matrices to form CS/SF/GNP nanocomposite membranes for ternary gas separation (CO_2_/N_2_/H_2_). GNPs created additional fast diffusion pathways and facilitated multi-mechanism synergy, enabling a CO_2_ permeability of 2126 GPU and a high selectivity of 104 and 52 with CO_2_/N_2_ and CO_2_/H_2_, respectively.

### 5.3. Resource Extraction

In the context of green and low-carbon development, membrane technologies are expanding from pollutant treatment toward selective resource recovery [204,205,206]. With directional permeation and enhanced mass transfer, low-carbon membranes can enable targeted enrichment of strategic metals (e.g., lithium, uranium, rare earth elements) and nutrients (e.g., nitrogen and phosphorus) from complex water bodies and salt lake brines [207,208,209]. The resource recovery process mainly includes impurity removal, pretreatment, concentration and enrichment, selective separation, and product purification [210,211,212]. Membrane separation technology is mainly applied in the concentration and separation stages. Under normal temperature and low pressure conditions, through the synergistic action of multiple mechanisms, the target ions can be extensively concentrated and separated [213].

In addition, the trade-off effect of membrane permeability and selectivity is currently a key scientific problem faced [214,215]. Regarding this current issue, there have been many studies on the interface design at the molecular level of membrane materials, and corresponding progress has been made [216,217]. Setiawan et al. [218] used the dry–wet spinning technology to prepare a complete PES hollow fiber membrane, and then successfully prepared a PES-IP-BOH membrane with a polyamide selective layer on the surface through the IP process. Experiments proved that the membrane had a rejection rate of over 96% for Na_2_SO_4_ and had little rejection for monovalent salts, thus demonstrating excellent separation selectivity for the first and second valence ions, up to 23. It indicated that interface design has good application potential in ion separation.

Mani et al. [219] modified bovine serum albumin (BSA) membranes via phosphorylation at the molecular interaction level, increasing hydration and ion-exchange capacity by approximately 30-fold—the membranes achieved a Cs^+^/Eu^3+^ selectivity of 28.23. Liu et al. [220] fabricated a titanium-based lithium-ion sieve (LIS) nanofiber membrane by electrospinning and calcination, and then prepared a PVB-HTO/PVDF dual-layer membrane by phase inversion. HTO nanofibers provided selective adsorption sites for Li^+^. In photothermal evaporation using Qarhan Salt Lake brine, the dual-layer structure enhanced adsorption, enrichment, and mass transfer dynamically. Despite a Mg^2+^/Li^+^ mass ratio of 249, a lithium recovery of 7.90 mg·g^−1^ was achieved with an ultra-high Mg^2+^/Li^+^ separation factor of 1168.

### 5.4. Desalination

With the increasing demand for freshwater resources, membrane separation technology plays a crucial role in the field of seawater desalination [208]. Low-carbon membrane materials such as biomass-derived membranes, covalent organic frameworks (COFs) membranes, and graphene-based membranes can maintain a high desalination rate while significantly reducing energy consumption and environmental impact [69,221,222]. The mechanism by which nanofiltration and reverse osmosis membranes retain salt is achieved through the synergy of pore size screening and charge effects [223]; however, nanofiltration membranes face trade-off effects and membrane fouling problems [224]. The operating pressure and energy consumption of reverse osmosis membranes are proportional to the salt content in the water and the target freshwater recovery rate. There are significant challenges in treating high concentrations of brine (>7.5 wt%), and the water flux during evaporation is low under low temperature conditions [225].

In response to this and based on a novel mass energy coupling concept, Zhao et al. [226] designed a light responsive COF membrane and a dual-drive system combining solar energy and a vacuum. This system could achieve a water flux of 120 kg·m^−2^·h^−1^ at a temperature of 30 °C, and a salt retention rate of over 99%. Moreover, it exhibited excellent and stable performance within a wide salinity range of 0.1–7.5 wt%, significantly reducing thermal energy consumption. Additionally, Shao et al. [217] used cellulose triacetate (CTA) as the base and designed a “homologous matching” strategy, introducing biomass-derived carbon quantum dots into the interface polymerization process of CTA and PA. They optimized the reaction kinetics and guided the formation of a thinner, denser, and more hydrophilic separation layer. This simultaneously increased the water flux of the reverse osmosis membrane (18.3 L·m^−2^·h^−1^) and the salt retention rate (99.1%). They successfully solved the trade-off problem between permeability and selectivity of traditional membrane materials.

## 6. Conclusions and Outlook

### 6.1. Conclusions

This review systematically summarizes the development of low-carbon membrane separation materials under the broader context of green transformation, covering recent progress in design concepts, separation mechanisms, catalytic coupling technologies, and application scenarios. Remaining challenges and prospective directions for implementing low-carbon membranes in separation unit operations are also discussed.

### 6.2. Outlook

Despite their promise for sustainable separation applications, low-carbon membrane materials face persistent challenges in balancing performance with environmental footprints, controlling costs, and achieving large-scale fabrication. Future research should prioritize the following directions:

(1) Data-driven assistance for the development and research of basic materials by utilizing machine learning and high-throughput screening techniques to establish a database of membrane materials, as well as virtual simulation to predict material experiments, thereby reducing the time and cost of research and development.
(2) Strengthen the top-level design and multi-functional integration of membrane materials by clarifying the principle of synergy between low-carbon membranes and catalytic technologies, enhancing the deep integration of separation and reaction processes and creating “separation-degradation-regeneration” integrated multi-functional membrane materials.
(3) Expand applications under extreme conditions and promote intelligent operation and maintenance. By optimizing the preparation process, adverse effects can be alleviated, such as reduced separation efficiency and the lifespans of membranes in harsh environments; moreover, sensors and digital control technologies can be combined to establish an integrated intelligent system with monitoring, early warning, and regulation functions, achieving intelligent management.

Ultimately, to enable green and sustainable closed-loop lifecycle for membrane materials can, performance trade-off bottlenecks must be overcome, persistent issues such as membrane fouling and limited service life should be alleviated, and the multiple constraints imposed by performance and cost considerations need to be fundamentally transcended.

## Figures and Tables

**Table 1:** Membrane categories, core advantages, and major limitations of low-carbon membrane materials.

Membrane Categories	Core Advantages	Major Limitations	Ref.
Low-carbon polymer membranes	Low cost Good processability Excellent flexibility Wide availability of raw materials Partially renewable or biodegradable	Poor long-term operational stability Moderate organic solvent resistance Moderate acid resistance Moderate alkali resistance Weak antifouling ability Intrinsic limitations in separation performance	[17,18,19,20]
Low-carbon 2D material membranes	High specific surface area High mechanical strength Excellent physicochemical properties Easy surface functionalization High separation precision.	High carbon footprint in raw materials and fabrication Susceptible to surface defects Challenges in scalable fabrication Susceptibility to interlayer swelling or stacking	[21,22,23,24]
Low-carbon mixed-matrix membranes	High mechanical strength Good stability Tunable structure and functionality Easy scalability Long membrane service life	Susceptibility to internal defects High cost for large-scale production	[25,26,27,28]
Low-carbon surface-modified membranes	Long membrane service life Low carbon footprint in modification process Enhanced antifouling and catalytic properties via targeted design	Detachment of modification layer during long-term operation Low permeability High cost of modification process Limited separation precision	[29,30,31,32,33]
Low-carbon ceramic membranes	Thermal and chemical stability High-temperature resistance Strong acid/alkali resistance High mechanical strength Recyclable raw materials	Low specific surface area High brittleness Low permeation flux Difficulty in large-scale production High energy consumption in sintering process	[34,35,36,37]
Low-carbon biochar membranes	Ultra-low carbon footprint Biodegradable raw materials Abundant porous structure Tunable surface hydrophilicity/hydrophobicity Low cost	Poor uniformity of pore structure Difficulty in scalable fabrication Low mechanical strength Poor high-temperature resistance Challenges in controlling separation precision	[38,39,40]
Low-carbon hollow fiber membranes	Ultra-high packing density Low operating pressure High modularity Easy scalability Continuous manufacturing process Self-supporting structure	Difficult to control selective layer defects Concentration polarization aggravates fouling Risk of fiber breakage Small inner diameter prone to fouling and clogging Difficult online detection and repair	[41,42,43]

## Data Availability

No new data were created or analyzed in this study. Data sharing is not applicable to this article.
